# Phylogeny and expression profiling of *CAD and CAD-like *genes in hybrid *Populus *(*P. deltoides *× *P. nigra*): evidence from herbivore damage for subfunctionalization and functional divergence

**DOI:** 10.1186/1471-2229-10-100

**Published:** 2010-05-28

**Authors:** Abdelali Barakat, Agnieszka Bagniewska-Zadworna, Christopher J Frost, John E Carlson

**Affiliations:** 1 The School of Forest Resources, and The Huck Institutes of the Life Sciences, Pennsylvania State University, 324 Forest Resources Building, University Park, PA 16802, USA; 2 Department of General Botany, Institute of Experimental Biology, Adam Mickiewicz University, Umultowska 89, 61-614 Poznań, Poland; 3 Center for Chemical Ecology, Pennsylvania State University, University Park, PA 16802, USA

## Abstract

**Background:**

Cinnamyl Alcohol Dehydrogenase (CAD) proteins function in lignin biosynthesis and play a critical role in wood development and plant defense against stresses. Previous phylogenetic studies did not include genes from seedless plants and did not reflect the deep evolutionary history of this gene family. We reanalyzed the phylogeny of *CAD *and *CAD-like *genes using a representative dataset including lycophyte and bryophyte sequences. Many *CAD/CAD-like *genes do not seem to be associated with wood development under normal growth conditions. To gain insight into the functional evolution of *CAD/CAD-like *genes, we analyzed their expression in *Populus *plant tissues in response to feeding damage by gypsy moth larvae (*Lymantria dispar *L.). Expression of *CAD/CAD-like *genes in *Populus *tissues (xylem, leaves, and barks) was analyzed in herbivore-treated and non-treated plants by real time quantitative RT-PCR.

**Results:**

*CAD family *genes were distributed in three classes based on sequence conservation. All the three classes are represented by seedless as well as seed plants, including the class of *bona fide *lignin pathway genes. The expression of some *CAD/CAD-like *genes that are not associated with xylem development were induced following herbivore damage in leaves, while other genes were induced in only bark or xylem tissues. Five of the *CAD/CAD-like *genes, however, showed a shift in expression from one tissue to another between non-treated and herbivore-treated plants. Systemic expression of the *CAD/CAD-like *genes was generally suppressed.

**Conclusions:**

Our results indicated a correlation between the evolution of the *CAD *gene family and lignin and that the three classes of genes may have evolved in the ancestor of land plants. Our results also suggest that the *CAD/CAD-like *genes have evolved a diversity of expression profiles and potentially different functions, but that they are nonetheless co-regulated under stress conditions.

## Background

Lignin is a phenolic heteropolymer that plays a central role in plant structure by providing rigidity and hydrophobicity to xylem cell walls [1], which facilitates the conduction of water and minerals throughout the plant body [2]. The lignin synthesis pathway involves many substrates and enzymes, among which the CAD enzyme catalyzes the last step in the synthesis of monolignol precursors. While mutations in a single real *CAD *gene rarely affect growth of transgenic plants, some natural mutants or double mutants have shown abnormal developmental and structural phenotypes. For instance, *Arabidopsis *plants with double mutations in the two major *CAD *genes associated with lignin biosynthesis (*AtCAD3 *and *AtCAD4*) present prostrate stems linked to weakness of the vasculature, as well as a reduction in the stem size and diameter [3]. The natural *brown midrib (bm) *mutants in sorghum and maize [4-6] which presented 20% reduction in lignin content [7,8], showed several phenotypic variations including increased lodging, reduced yield, re-growth, height, variation in the flowering time, and tillering in some environments, compared to wild type counterparts.

Lignin plays an important role in plant defense against insects and pathogens [9-12]. Lignin functions as a physical impediment, either physically blocking pathogen entry or increasing leaf toughness to make chewing by herbivores more difficult. While lignin synthesis is a central component of most plant growth, the rate of lignin synthesis can be accelerated by herbivory or pathogen attack. The rapid deposition of lignin or lignin-like materials may inhibit further growth and confine the invading pathogen or reduce the fecundity of an herbivore [13-18]. For instance, infection of *Populus *with the rust fungus, *Melampsora larici-populina*, led to a strong accumulation of monolignols within two days of infection [19]. Similarly, Norway spruce bark and cambium inoculated with the bark beetle associated fungal pathogen, *Ceratocystis polonica*, became partially or completely lignified surrounding the inoculation site [20,21]. Lignin functions in plant defense against herbivores by increasing leaf toughness and decreasing leaf nutritional content [22]. Moreover, lignin production can be altered in response to herbivore feeding [23]. Considering the importance of lignin in plant defense and the effect of herbivory on the phenylpropanoid pathway [24-29], it is not surprising that previous studies have shown an increase in the expression of *CAD/CAD-like *genes in plants infected with pests and pathogens [30-32].

In *Populus*, we reported 15 *CAD/CAD-like *genes distributed in three main classes [33]. However, our phylogeny in that study [33] did not include sequences from seedless plants and could not draw clearly the complete evolutionary history of this gene family. Among the *CAD/CAD-like *genes in *Populus*, only two (*PoptrCAD4 *and *PoptrCAD10*) have been shown to be preferentially expressed in xylem under normal growth conditions. In contrast, three *Populus *genes (*PoptrCAD7, PoptrCAD12*, and *PoptrCAD13*) are preferentially expressed in leaves, and one gene is preferentially expressed in both leaves and xylem (*PoptrCAD9*). The functions of *Populus CAD/CAD-like *genes, except *PoptrCAD4 *and *PoptrCAD10*, are still unknown. Phylogenetic analysis has shown that all the *bona fide *lignin pathway *CAD *genes studied so far belong to Class I [33]. *Populus Sinapyl Alcohol Dehydrogenase *(*SAD*) genes [33,34] as well as *Arabidopsis AtCAD4*, *AtCAD6*, and *AtCAD5 *genes, which have been shown to be associated with lignin biosynthesis [35], were distributed in Class II. We do not know whether the distribution of *CAD/CAD-like *genes in three main classes reflects a functional divergence and several questions related to the phylogeny and the function of *CAD/CAD-like *genes remain to be answered. For instance, are Class I genes associated only with xylem development while Class II and Class III genes function in defense against biotic stresses? Do genes from the three classes compensate for each other both under normal growth and stress conditions? Will different *CAD/CAD-like *genes respond similarly to herbivore stress? Will herbivore-mediated expression of the *CAD/CAD-like *gene family members differ between physically damaged tissue and systemic, undamaged tissue?

To address these questions, we re-analyzed the phylogeny of *CAD/CAD-like *genes using a dataset that includes sequences from a moss and a lycophyte. We also used quantitative RT-PCR to analyze the expression of ten *CAD/CAD-like *genes in several tissues (leaves, bark, xylem systemic, and undamaged leaf tissue) from hybrid *Populus *plants challenged with gypsy moth larvae and unchallenged control plants. These studies permitted us to relate the evolution of lignin and the CAD family, and to get insight into the evolution of gene functionalization within the family.

## Results

### Phylogeny of *CAD/CAD-like *genes

**Figure 1:**
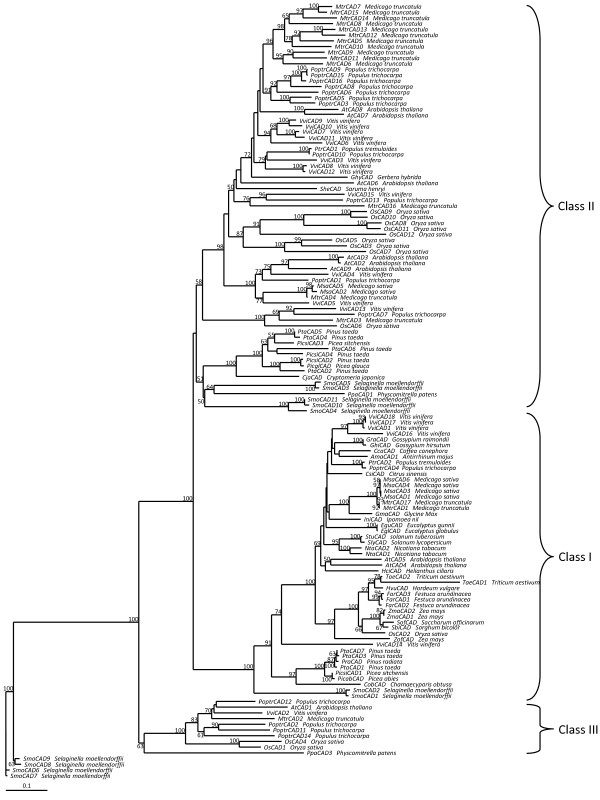
**Phylogenetic tree of *CAD/CAD-like *genes**. Numbers above branches refer to bootstrap values. Values that were below 50 were not reported. Brackets highlight the three classes of *CAD/CAD-like *genes.

Phylogenetic analysis was conducted using maximum likelihood with protein sequences from over 40 species belonging to various land plant lineages (see Additional file 1 Table S1). The analysis showed that *CAD/CAD-like *genes are distributed in three major classes (Fig. 1). The distribution of genes in Class I and III are supported with good bootstrap values (100 and 63 for Class I and Class III respectively), while Class II is not as well supported (bootstrap value = 49). Class I (*bona fide *lignin gene class) and Class III *CAD/CAD-like *genes include sequences from angiosperm and moss (*Physcomitrella*). However, Class III is represented by sequences from angiosperms and moss but not the lycopod *Selaginella*.

In Class I, II, and III, genes from seedless plants cluster separately from the angiosperms. The distribution of *CAD/CAD-like *genes in the tree shows evidence of several plant-specific or lineage specific duplications. Class III includes few genes from both monocots and eudicots but no gymnosperm sequences. In all three classes, sequences from monocots, eudicots, gymnosperms, and seedless plants cluster separately indicating that events that contributed to the duplication of these genes have occurred after the splits of those lineages from each other.

### Expression profiling of *CAD *genes in herbivore-damaged tissues

**Figure 2:**
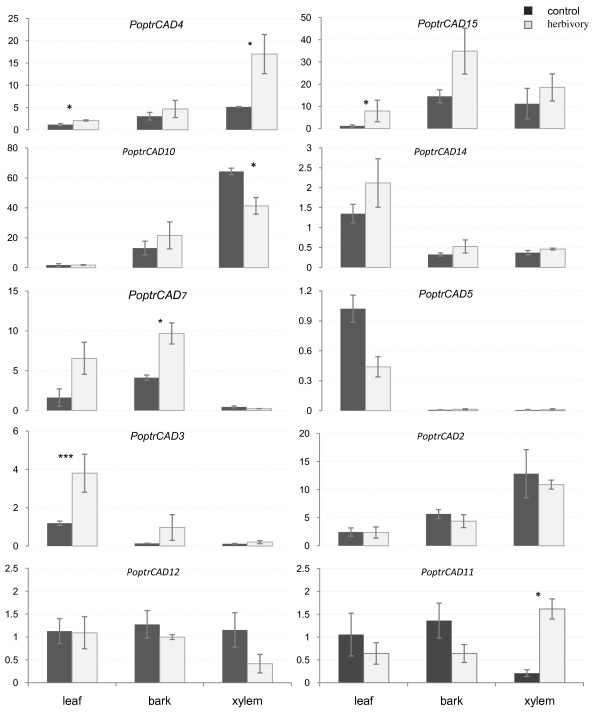
**Expression of *Populus CAD/CAD-like *genes**. Relative quantification of expression was analyzed in different tissues from non-treated (control) *vs*. herbivore-treated leaves. The name of each gene is indicated at the top of each histogram. Tissues studied are shown at the bottom of the diagrams. ± means SE of three biological replicate samples; *p < 0.05, **p < 0.01, ***p < 0,001 according to Student's t-test. Y axis indicates the relative expression level of each gene compared to the control tissue (leaves).

In *Populus*, the *CAD/CAD-like *gene family is represented by 15 genes [33]. However, only two genes (*PoptrCAD4 *and *PoptrCAD10*) are preferentially expressed in xylem, where lignin deposition is required to increase plant tensile strength. Most of the remaining genes are induced in leaves and/or bark [33]. To determine which of the *Populus CAD/CAD-like *genes are associated with response to herbivory, we analyzed the expression of 10 *CAD/CAD-like *genes using quantitative RT-PCR in various tissues from plants challenged with *Lymantria dispar*. To address questions related to the evolution of *CAD/CAD-like *genes, we selected ten genes representing all phylogenetic clusters in the three classes of *CAD/CAD-like *genes and presenting different expression patterns in non-stressed plants. We searched for a local response by analyzing the expression in tissues (leaves, bark, and xylem) directly in contact with the herbivores, even though gypsy moth only feed on the foliage. Real time RT-PCR experiments showed that six of the ten *CAD/CAD-like *genes studied exhibited a preferential expression in at least one of the tissues analyzed (Fig. 2). The expression profiles of *CAD/CAD-like *genes in tissues from stressed plants can be divided in five groups: The group 1 genes (*PoptrCAD4 *and *PoptrCAD11*) showed a preferential expression in xylem. The group 2 genes (*PoptrCAD4, PoptrCAD3*, and *PoptrCAD15*) showed an induced expression in leaves. The only gene in group 3 (*PoptrCAD7*) is highly induced in the bark. Group 4 genes (*PoptrCAD2, PoptrCAD5, PoptrCAD12*, and *PoptrCAD14*) showed similar expression profiles in all tissues from stressed and control plants, and thus appear to be constitutively expressed. Finally, the expression of the one gene in Group 5 (*PoptrCAD10*) is suppressed in xylem.

While *PoptrCAD4 *from group 1 was preferentially expressed in xylem, *PoptrCAD11 *showed similar expression levels in xylem, bark, and leaves under normal growth conditions [33]. The expression of *PoptrCAD11 *and *PoptrCAD4 *at least doubled in xylem tissues challenged with *Lymantria dispar*. *PoptrCAD4 *and *PoptrCAD10*, which are associated with lignin biosynthesis during development, present different expression patterns under herbivore stress. While *PoptrCAD4 *is highly induced in xylem from stressed plants, *PoptrCAD10 *showed a lower level of expression in treated plants. *PoptrCAD7*, which showed a preferential expression in the leaf under normal growth conditions, changed the location (shifted) of its preferential expression to the bark under herbivory conditions. *PoptrCAD2, PoptrCAD5, PoptrCAD12 *and *PoptrCAD14 *did not show any expression difference in tissues from stressed and non-stressed plants. One-way ANOVA results showed a large variation in gene expression between tissues in non-treated plants (except genes *PoptrCAD2*, *PoptrCAD11*, *PoptrCAD12*, and *PoptrCAD15*) and herbivore treated plants (except *PoptrCAD12 *and *PoptrCAD15*).

### Expression profiling of *CAD *genes in systemic leaves

**Figure 3:**
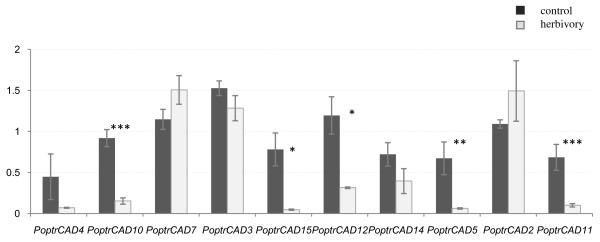
**Relative expression of *CAD/CAD-like *genes in directly treated *vs *systemically induced leaves from *Populus *plants**. ± means SE of three biological replicate samples.; *p < 0.05, **p < 0.01, ***p < 0,001 according to Student's t-test. Y axis indicates the relative expression level of each gene compared to the control tissue (non-treated leaves).

**Figure 4:**
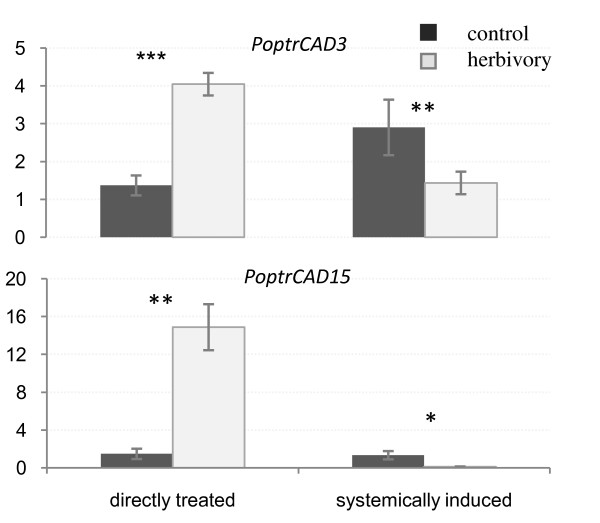
**Relative expression quantification of *PoptrCAD3 *and *PoptrCAD15 *genes in directly treated *vs *systemically induced leaves from *Populus *plants**. Leaves from the corresponding tissue of control plants were used as a calibrator. ± means SE of three biological replicate samples.; **p < 0.01, ***p < 0,001 according to Student's t-test. Y axis indicates the relative expression level of each gene compared to the control tissue (non-treated leaves).

To test if the differential expression of *CAD/CAD-like *genes shown in *Populus *plants challenged with herbivores was systemic, we analyzed the expression in leaves that were not in contact with herbivores (systemic leaves) (Fig. 3). Five of the ten *CAD/CAD-like *genes (*PoptrCAD10, PoptrCAD15*, *PoptrCAD12*, *PoptrCAD5*, and *PoptrCAD11) *were significantly down-regulated in the systemic leaves. The other five genes, *PoptrCAD2, PoptrCAD3, PoptrCAD4, PoptrCAD7 *and *PoptrCAD14*, were not affected by herbivory in systemic leaves. To compare the response of *CAD/CAD-like *genes between directly treated and systemic leaves, we analyzed the expression level of two different genes (*PoptrCAD3 *and *PoptrCAD15*). The analysis showed that the expression of *PoptrCAD3 *and *PoptrCAD15 *was higher in the directly treated leaves from stressed plants compared to control plants (Fig. 4). However, the expression of these two genes was very low in systemic leaves from stressed plants compared to control plants.

### Expression of the *rubisco *gene in directly treated and systemically induced tissues

**Figure 5:**
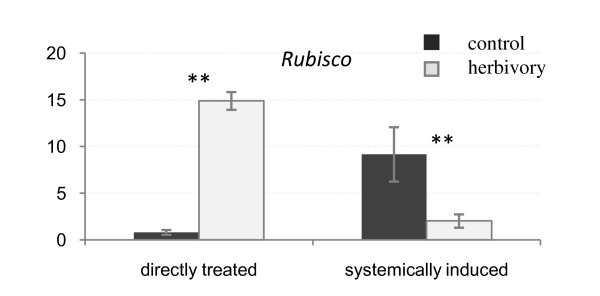
**Relative expression of the *rubisco *gene in directly treated and systemically induced *Populus *leaves**. Leaves from the corresponding tissue of control plants were used as a calibrator. ± means SE; **p < 0.01, ***p < 0,001 according to Student's t-test. Y axis indicates the relative expression level of each gene compared to the control tissue (non-treated leaves).

Plants tend to redirect their metabolism in response to herbivory in ways that affect both the damaged and undamaged parts of the plant [36,37]. To check whether the low expression level of *CAD/CAD-like *genes observed in non-treated tissues was linked to an overall lower metabolism in those tissues, we analyzed the expression of the *rubisco *gene, as *rubisco *and other photosynthetic genes are known to be suppressed by herbivory [38-42]. In our experiment, the expression of the *rubisco *gene was induced in directly treated leaves and was repressed in the systemic undamaged leaves (Fig. 5).

## Discussion

Phylogenetic analysis of *CAD/CAD-like *genes published recently by our group [33] used a sequence dataset that did not include seedless plant sequences. This limited insights into the evolution of *CAD/CAD-like *genes in relationship with the evolution of lignin in land plants. Here we re-analyzed the phylogeny of *CAD/CAD-like *genes using sequences from the ten plants for which the genomes are completely sequenced including the moss *Physcomitrella *and the lycopod *Selaginella*. Phylogenetic analysis showed that *CAD/CAD-like *genes, except four *Selaginella *sequences (*SmoCAD6*, *SmoCAD7*, *SmoCAD8*, and *SmoCAD9*) are distributed in three classes. This resulting tree is in agreement with our previous results [33], however the origin of all classes of *CAD/CAD-like *genes in seed plants from genes previously residing in mosses is now documented. The four *Selaginella *sequences that do not cluster with the other genes could correspond to other dehydrogenase genes that are highly similar to *CAD/CAD-like *genes. Two of the three classes (Class I and Class III) are well supported while Class II is not. Class I and Class II include genes from both *Physcomitrella *and *Selaginella*. This indicates that the evolution of these two classes happened in the ancestor of land plants. Class III includes a sequence from *Physcomitrella *which is the most basal among the Class III genes, but neither *Selaginella *nor gymnosperm sequences. This suggests that Class III may represent the first *CAD *genes that evolved with or after the divergence of mosses from lycophytes. The lack of gymnosperm sequences in this class either represents gene loss following divergence of Classes I and II, or is perhaps an artifact of the current incomplete coverage of the transcriptome of gymnosperm plants. The distribution of *bona fide CAD *genes (Class I) in a monophyletic clade including sequences from mosses, lycophytes, gymnosperms, and angiosperms is an indication that the real *CAD *genes existed in the ancestor of land plants and their evolution is intimately associated with the evolution of lignin. This is in agreement with previous studies showing that *Selaginella *has real lignin [43]. Other studies have reported lignin in red alga *Calliarthron cheilosporioides *[44], but it was suggested that this was the result of convergent evolution. Class II and Class III corresponding *CAD-like *genes seems to be the result of duplications that had happen in the ancestor of land plants. Some of these genes still function in lignin biosynthesis under normal growth conditions as was exemplified by *PoptrCAD10 *and *AtCAD6 *[33,35]. The other *CAD-like *genes may function in lignin biosynthesis under various stress conditions or have evolved different functions. For instance, a compilation of *Populus *gene expression data from the PopGenIE database [19,45,46] showed that several *CAD/CAD-like *genes (*PoptrCAD5*, *PoptrCAD7*, *PoptrCAD14*, and *PoptrCAD12*), as well as others not studied here (*PoptrCAD6*, *PoptrCAD13*, and *PoptrCAD1*), were found to be induced under biotic (rust infection) and abiotic stresses (mechanical damage, drought, ozone, UV-B, and elevated CO2). Furthermore, it was suggested that the *Quercus QsCAD1 *gene functions in plant defense against *Phytophthora cinnamomi *by deactivation of toxins produced by this pathogen [32]. Whatever the nature of defense mechanisms they belong to, the phylogenetic distribution of *CAD-like *genes suggests that the evolution of these mechanisms had occurred in the ancestor of land plants, which seems to be already targets of pests and pathogens back at that time.

A recent study [33] showed that within the 15 *CAD/CAD-like *genes from *Populus trichocarpa*, only *PoptrCAD4 *and *PoptrCAD10 *genes are preferentially expressed in xylem and seem to be associated with wood development. The other *CAD/CAD-like *genes present various expression profiles and most are induced in leaves or bark. It has been suggested that these genes are involved in defense against plant pathogen attack. For instance, plant treatments with [sulphinyl] acetic acid, 1.1 dimethyl ester (OH-PAS), a specific CAD inhibitor, reduced the penetration resistance of barley to *Puccinia hordei*. It has been also suggested that some *CAD/CAD-like *genes such as *QsCAD1 *from *Quercus suber *are potentially involved in deactivation of toxins produced by phytopathogens [32]. Other studies [47-51] reported a correlation between plant infections by pathogens or herbivore attack and either the amount of lignin or an increase in the expression of lignin pathway associated genes including *CAD/CAD-like *genes. In this study, we analyzed the expression of 10 of the 15 *CAD/CAD-like *genes in various tissues of *Populus *plants challenged with *Lymantria dispar *and that *CAD/CAD-like *genes present various expression profiles indicating a highly probable functional divergence of genes within this family. Six of the genes studied showed preferential expression in at least one tissue from the stressed plants, suggesting that *CAD/CAD-like *genes act together in defending the plant against pathogen attack. Genes from functional group 1 (*PoptrCAD4, PoptrCAD11*) and group 3 (*PoptrCAD*7) showed differential expression in xylem and bark, respectively. *PoptrCAD4 *and *PoptrCAD11 *share promoter motifs involved in response to methyl-jasmonate (MeJa), abscisic acid (ABA), and wounding [33]. *PoptrCAD*7 possess several motifs including the ones involved in MeJa and wound response [33]. This gene was previously reported as having been induced following MeJa, wounding, rust infection, and UV treatments [19,45,46]. The induction of those genes in the bark and xylem could be linked to the extensive damage caused to the leaves by the herbivore. It is highly probable that after losing a large fraction of their leaves, the plants respond by protecting the main stem. Genes from group 2 (*PoptrCAD3, PoptrCAD4 *and *PoptrCAD15*) showed preferential expression in leaves from herbivore-stressed plants. *PoptrCAD4 *also showed preferential expression in xylem in stressed plants. Promoter sequence analysis, showed that these three genes shared several motifs including some involved in response to defense against biotic and abiotic stress such as MeJA and ABA [52-55]. This is also in accordance with results from previous studies [19,45,46] showing that *poptrCAD4 *was induced under MeJA, mechanical damage, drought, UV-B, ozone treatments and rust infection. *PoptrCAD15 *was shown to be induced under drought and elevated CO2. The constitutive expression of those genes in leaves under normal conditions and their increase in expression in damaged leaves and stems suggests that these genes play an important role in defense against herbivore attack, or at the very least are responsive to herbivore stress.

*PoptrCAD2, PoptrCAD5, PoptrCAD12 *and *PoptrCAD14 *did not show any difference in expression between tissues from stressed and non-stressed plants. These genes present similar motifs as the ones preferentially expressed in tissues from stressed plants. These motifs are involved in response to MeJA, ABA, wounding, and fungal elicitors. The similar expression profile in various tissues between non-stressed and stressed tissues despite the presence in their promoter sequences of several motifs involved in response to biotic and abiotic stresses indicates that those genes may be involved in response to other types of stress than herbivory. This assumption is in accordance with previous results showing that *PoptrCAD12 *and *PoptrCAD14 *were induced following MeJA treatment, drought, and elevated CO2 [45,46].

Three of the ten genes (*PoptrCAD3, PoptrCAD11 *and *PoptrCAD15) *analyzed were induced in herbivore-stressed plants (Table 1). *PoptrCAD3 *and *PoptrCAD15 *genes, which showed similar expression among tissues in the non-stressed plants, were induced in leaves of treated plants. A similar situation was shown for *PoptrCAD11*, which was induced in the xylem of treated plants. Two genes (*PoptrCAD4 *and *PoptrCAD7*) showed a shift in expression profiles between non-stressed and herbivore stressed plants (Table 1). *PoptrCAD4*, which showed preferential expression in xylem in non-stressed plants, was induced upon herbivory in both xylem and leaves. *PoptrCAD7*, which was preferentially expressed in leaves in non-stressed plants, showed a high level of expression in the bark of treated plants. These results show that plants induce upon stress the expression of *CAD-like *genes that are expressed at only low levels during normal plant development. The results also suggest that the expression of other genes such as *bona fide *lignin biosynthesis genes may shift expression (change specificity) to other tissues to defend against pathogens. These results rule against the hypothesis of absolute functional divergence between *bona fide CAD *genes and *CAD-like *genes. It is likely that genes from the three classes are still involved in lignin biosynthesis but have evolved specialized expression profiles.

**Table 1:** List *CAD/CAD-like genes *induced or suppressed in gypsy moth stressed plants.

Gene	Normal Growth			Stressed		
	
	Leaf	Bark	Xylem	Leaf	Bark	Xylem
*PoptrCAD4*			I		SI	I

*PoptrCAD11*						SI

*PoptrCAD2*			I			I

*PoptrCAD10*			I			SR

*PoptrCAD3*				SI		

*PoptrCAD15*				SI		

*PoptrCAD7*	I				SI	

I: Expression induction; SI: shift and induction of expression among tissues. SR: shift and repression of expression among tissues.

Phylogenetic analyses of *Populus CAD/CAD-like *genes showed that *PoptrCAD2 *and *PoptrCAD11 *are paralogs. In this study we observed that these two genes present a striking difference in their expression profiles in stressed plants (Table 1). *PoptrCAD11 *is highly induced in xylem from stressed plants compared to *PoptrCAD2*. In non-stressed plants, however, both genes showed similar expression patterns in the tissues analyzed [33]. A similar situation was observed for the paralogs *PoptrCAD3 *and *PoptrCAD5*. While *PoptrCAD5 *presented similar expression profiles between non-stressed and stressed plants, *PoptrCAD3 *was increased in expression in leaves of stressed plants. The expression profiles suggest that the duplication which created the *PoptrCAD3 *and *PoptrCAD5*, and the *PoptrCAD2 *and *PoptrCAD11 *gene pairs has been followed by a diversification in expression.

Comparison of gene expression in damaged and systemic leaves showed a down regulation of most *CAD/CAD-like *genes in systemic leaves from stressed plants compared to their counterparts from non-stressed plants. This phenomenon may be linked to a re-allocation of plant resources that has been previously reported, in which the increase in resource utilization for the defense of damaged leaves translates into a slower general metabolism in non-damaged parts of the plant [36,37]. Our result of differential expression of the *rubisco *gene, as a marker for primary metabolism, in non-treated leaves both in stressed and non-stressed plants, is suggestive that such reallocation of resources is occurring in poplar in response to herbivory, which could help explain the suppressed CAD expression levels systemically in stressed plants. This also indicates that future studies should include the collection of metabolic data to evaluate resource reallocation to confirm if the low expression of *CAD/CAD-like *genes in non-treated tissues reflects a redirection of metabolic flux to the damaged tissues rather than systemic induction of defense genes, rather than a lack of a systemic defense response.

## Conclusions

In conclusion, we confirmed the distribution of *CAD/CAD-like *genes in plants into into three phylogenetic classes that evolved in the ancestor of land plants. We have shown that the expression of genes in the *CAD/CAD-like *family is coordinated in tissues under both normal growth and under biotic stress conditions. Some *CAD/CAD-like *genes in *Populus *that do not function in lignin biosynthesis in xylem may function together in defense against pest attack. We demonstrated that *Populus CAD/CAD-like *genes may play their role in defense in a complex tissue-specific manner. We showed that duplicate *CAD/CAD-like *genes have evolved different expression profiles and may have or be evolving towards modified functions as well. Finally, these results take us a step closer to understanding functional divergence among *CAD/CAD-like *genes, a key gene family in the evolution of land plants.

## Methods

### Plant material

**Figure 6:**
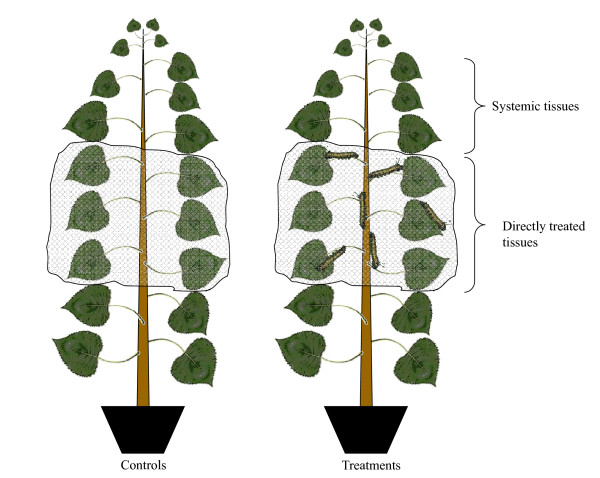
**Treatment scheme for the placement of *Lymantria dispar *larvae on *Populus *plants**.

Leaves, bark, and stem secondary xylem were collected from young hybrid *Populus *OGY (*P. deltoides *× *P. nigra*) trees grown in a culture chamber at 25°C and 18°C during the day and night, respectively. The plants were grown at 16 h/8 h day/night light regime and at 60% humidity. For herbivore treatments, we enclosed 10 gypsy moth (*Lymantria dispar*) larvae within mesh bags that contained leaves from LPI 8-15 (Fig. 6). Control plants were bagged on the same position but without insect larvae. Tissues were harvested from directly treated damaged and systemic (non-damaged three top rows of leaves) after 24 hours herbivory. Tissues were frozen in liquid nitrogen and stored at -80°C until use.

### Sequences alignment and phylogenetic analyses

Sequences used in the phylogenetic analysis include the dataset from [33] to which we added sequences from the genomes of *Physcomitrella patens *and *Selaginella moellendorffii *retrieved from JGI [56]. Sequence alignments and phylogenies were constructed as previously described [33].

### RNA isolation and cDNA synthesis

Total RNA was isolated using a CTAB method [57] with minor modifications. The RNA quality and concentration was assessed using an Agilent 2100 Bioanalyzer (Agilent Technologies). To remove any contaminating genomic DNA, RNA samples were treated with RNase-free DNase (Applied Biosystems) before RT-PCR experiments were conducted. RNA was reverse transcribed using random primers from the High Capacity cDNA Reverse Transcription kit (Applied Biosystems) following the manufacturer's recommendations. One microgram of total RNA from each sample was reverse-transcribed for each single RT-PCR reaction.

### *CAD/CAD-like *gene expression analysis using quantitative real time RT-PCR

Quantitative real time PCR reactions were performed as described previously [33]. In summary, reactions were prepared using the SYBR Green Master Mix kit (Applied Biosystems) and performed in an Applied Biosystems 7500 Fast Real-Time PCR system (Applied Biosystems) with default parameters. Primers used in this study were designed using Primer Express^® ^software (Applied Biosystems). We used the gene encoding the 18 S rRNA as an endogenous control for normalization for template quantity. Dissociation curves were used to verify the specificity of PCR amplification. Expression analyses were performed on three biological replicates (three trees) and three experimental replicates. Data was evaluated using the 7500 Fast System SDS software procedures (Applied Biosystems). Statistical analyses were performed using Statistica 6.0 software (StatSoft Poland Inc., Tulsa, OH, USA).

## List of abbreviations used

CAD: Cinnamyl alcohol dehydrogenase; SAD: Sinapyl Alcohol Dehydrogenase; nt: nucleotide; AA: amino acid; RT-PCR: Reverse transcriptase polymerase chain reaction.

## Authors' contributions

AB planned the project; designed the experiments, ran the phylogenetic analysis, contributed to the RT-PCR experiments, supervised the work of ABZ and wrote the manuscript. ABZ conducted the expression analyses. CF set up the herbivore stress experiment and assisted in revising the manuscript. JEC provided the financial support, contributed to the discussion of the research plans and results and helped in editing the manuscript. All authors read and approved the final manuscript.

## Supplementary Material

Additional file 1 **List of plant genes used in CAD gene phylogenetic analyses**. The gene names used in this study, the accession number, species, the database source, and names of previously published genes are indicated. VvGDB, *Vignus vinifera *genome database; MTGSP, *Medicago truncatula *genome sequencing project. Click here for file
